# Pituitary stalk interruption syndrome

**DOI:** 10.11604/pamj.2023.44.144.34585

**Published:** 2023-03-23

**Authors:** Krittin Ousirimaneechai, Thiti Snabboon

**Affiliations:** 1 Department of Medicine, Faculty of Medicine, Chulalongkorn University, Bangkok, Thailand,; 2 Excellence Center in Diabetes, Hormone and Metabolism, King Chulalongkorn Memorial Hospital, Thai Red Cross Society, Bangkok, Thailand

**Keywords:** Hypopituitarism, posterior pituitary ectopia, stalk interruption

## Image in medicine

A 22-year-old man presented with short stature and delayed puberty. He was born a healthy newborn and has achieved normal developmental milestones. The patient sometimes complained of lethargy and cold intolerance, which responded well to symptomatic treatment. His height at presentation was 145 cm (below the 3^rd^ percentile) with a BMI of 18.5 kg/m^2^. His external genitalia was a Tanner stage II with small testes bilaterally. The hormonal test showed anterior pituitary hormone deficiencies and his karyotype was 46, XY. MRI study revealed typical pituitary stalk interruption syndrome (PSIS) features. His height and sexual development responded well to hormonal replacement. PSIS is a rare congenital disorder of hypopituitarism with an incidence of 0.5/100,000 live births. Its etiology is still elusive with less than 10% of genetic predisposition. Classical triad MRI findings are characterized by markedly thin or absent pituitary stalk, ectopic posterior pituitary or bright spot position, and hypoplasia or aplasia of the anterior pituitary lobe within a small sellar fossa. The ectopic bright spot may be found anywhere along the infundibular axis, with commonly reported at the level of an infundibular recess of the third ventricle. Clinical presentation is heterogeneous depending on either isolated or multiple anterior pituitary hormone deficiencies, extra-pituitary malformations especially midline structure defects, and the age of diagnosis. Growth hormone deficiency and hypogonadism are the most common presentation, whereas posterior pituitary function is intact. Early recognition and timely hormonal replacement are pivotal roles in improving clinical outcomes.

**Figure 1:**
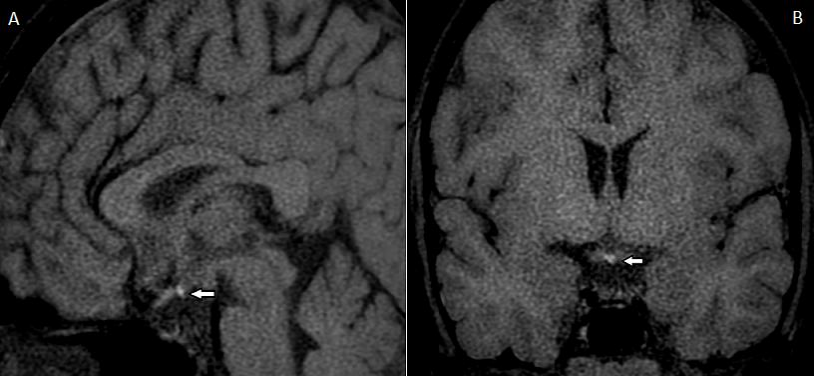
MRI pituitary gland, T1-weight non-contrast sagittal: A) and coronal view; B) showing a shallow sella turcica with the hypoplastic pituitary gland, interrupted pituitary stalk, and an ectopic bright spot site at the infundibular recess (arrow)

